# Refractory Prinzmetal Angina With Severe Right Coronary Artery Vasospasm and Bradycardia in a 46-Year-Old Female: A Complex Case Associated With Hypophosphatemia

**DOI:** 10.7759/cureus.100093

**Published:** 2025-12-25

**Authors:** Abbas Rachid, Suzan Iskandar, Hayab Karaki, Malek Mohammed, Hasan Kazma

**Affiliations:** 1 Internal Medicine, Lebanese University Faculty of Medicine, Beirut, LBN; 2 Cardiology, Lebanese University Faculty of Medicine, Beirut, LBN; 3 Medicine, Lebanese University Faculty of Medicine, Beirut, LBN; 4 Cardiology, Bahman University Hospital, Beirut, LBN

**Keywords:** angina pectoris, coronary vasospastic angina, electrolyte disturbances, refractory angina, symptomatic hypophosphatemia

## Abstract

Prinzmetal (vasospastic) angina is an uncommon cause of transient ST-segment elevation and potentially life-threatening arrhythmias, typically occurring at rest. Identification of precipitating factors, including metabolic and electrolyte disturbances, is critical for targeted management. We report the case of a 46-year-old female smoker with a family history of coronary artery disease and prior non-obstructive coronary angiography who presented with recurrent nocturnal chest pain radiating to the left arm and associated with diaphoresis. Initial electrocardiography demonstrated transient 1-mm ST-segment elevation in lead II, which resolved with anti-ischemic therapy. Two hours after discontinuation of intravenous nitroglycerin due to hypotension, the patient developed recurrent chest pain with 3-mm ST-segment elevation in the inferior leads and sinus bradycardia. Urgent coronary angiography revealed severe right coronary artery spasm, promptly relieved by intracoronary nitroglycerin. Subsequent laboratory evaluation identified significant hypophosphatemia (1.6 mg/dL) and vitamin D deficiency. Phosphate replacement was initiated, after which no further vasospastic episodes occurred. Hypophosphatemia may promote coronary vasospasm through impaired myocardial energy metabolism and endothelial dysfunction. This case highlights the importance of clarifying the temporal relationship between electrolyte abnormalities and vasospastic events and recognizing hypophosphatemia as a possible reversible contributor to refractory vasospastic angina. Severe right coronary artery spasm can closely mimic acute coronary syndrome and result in hemodynamic instability; prompt angiographic diagnosis, intracoronary vasodilator therapy, and correction of underlying metabolic disturbances are essential for effective management.

## Introduction

Acute myocardial infarction (AMI) most commonly results from obstructive coronary artery disease (CAD), defined as ≥50% stenosis on coronary angiography (CAG). However, approximately 1-14% of AMI cases occur in the absence of obstructive CAD, a clinical entity now recognized as myocardial infarction with non-obstructive coronary arteries (MINOCA) [1,2]. Beyond plaque rupture and thrombosis, alternative mechanisms, including coronary vasospasm, microvascular dysfunction, and endothelial impairment, are increasingly recognized contributors to myocardial ischemia and infarction [3].

Vasospastic angina (VSA), also known as Prinzmetal angina, is a distinct clinical entity characterized by anginal episodes at rest, transient ST-segment elevation, and preserved exercise tolerance. It can present in diverse scenarios, including acute coronary syndromes (ACS), arrhythmias, syncope, or even sudden cardiac death [1]. Although its pathophysiology is multifactorial, proposed mechanisms include epicardial and microvascular spasm, impaired nitric oxide-mediated vasodilation, endothelial dysfunction, increased vasoconstrictor activity, and oxidative stress [1,4].

We report a case of refractory Prinzmetal angina with an electrolyte disturbance, underscoring the importance of considering metabolic factors in the evaluation and management of VSA.

## Case presentation

We present the case of a 46-year-old female, an active smoker with a family history of CAD, who had a prior history of angina five years earlier. At that time, coronary angiography revealed a 40% stenosis in the proximal segment of the obtuse marginal branch (OM1). She was subsequently managed with verapamil, trandolapril, aspirin, atorvastatin, and isosorbide mononitrate. Medication adherence was reported by the patient and was objectively verified. Despite ongoing antianginal therapy, she continued to experience recurrent episodes of angina, predominantly at rest, consistent with refractory vasospastic angina.

**Figure 1:**
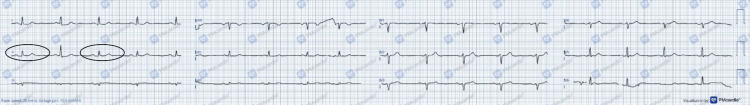
Admission electrocardiogram demonstrating 1 mm ST-segment elevation in lead II (blue arrows). Twelve-lead ECG converted using the PMcardio application.

She presented to the emergency department with nocturnal-onset, severe, compressive chest pain radiating to the left arm, associated with diaphoresis. On arrival, her electrocardiogram (ECG) demonstrated 1 mm ST-segment elevation in lead II. Given the initial concern for ACS, she received morphine, a loading dose of ticagrelor, atorvastatin, and enoxaparin. Intravenous nitroglycerin infusion was promptly initiated. Aspirin was deliberately withheld due to the clinical suspicion of vasospastic (Prinzmetal) angina as a potential alternative diagnosis to acute myocardial infarction. The ST-segment changes subsequently resolved (Figure 1).

**Figure 2:**
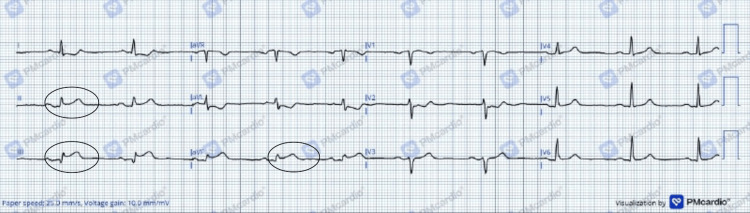
ECG of the patient demonstrating ST-segment elevation in inferior leads (II, III, aVF: black circles). Twelve-lead ECG of our patient converted using PMcardio application.

Two hours after discontinuation of IV nitroglycerin due to hypotension, the patient experienced recurrent chest pain. A repeat ECG revealed 3 mm ST-segment elevation in leads II, III, and aVF, with reciprocal ST depression in leads I and aVL (Figure 2), sinus bradycardia (HR=59), and associated dizziness.

**Figure 3:**
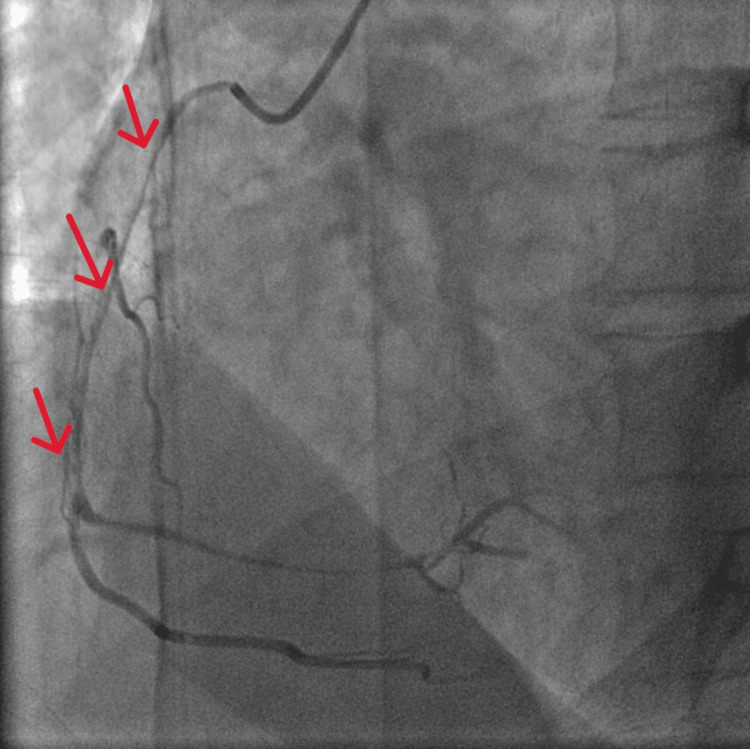
Coronary angiography demonstrating severe RCA spasm (red arrows). RCA: Right Coronary Spasm

Given concern for an acute inferior ST-elevation myocardial infarction, an urgent coronary angiography was performed. The angiogram revealed severe spasm of the right coronary artery (RCA) (Figure 3), which was promptly relieved with the administration of 1 mg intracoronary nitroglycerin (Video 1). She was restarted on IV nitroglycerin and received norepinephrine support as needed for hypotension.

**Video 1:** Resolution of right coronary artery spasm after intracoronary nitroglycerin administration.

During hospitalization, she developed recurrent episodes of bradycardia, though her vital signs remained stable otherwise. Physical examination was notable for generalized body aches and exertional fatigue, but there were no findings of localized limb edema or ongoing diaphoresis.

Laboratory evaluation demonstrated elevated high-sensitivity troponin I (3.214 ng/mL), consistent with myocardial injury. Notably, hypophosphatemia was identified in association with elevated parathyroid hormone levels and vitamin D deficiency, suggesting secondary hyperparathyroidism. Alkaline phosphatase remained within the normal range (104 U/L), indicating the absence of markedly increased bone turnover at this stage. Renal function and other electrolytes were within normal limits (Table 1). Phosphate and vitamin D supplementation were initiated in addition to optimization of antianginal therapy.

**Table 1:** Laboratory Investigations in our patient during hospitalization. WBC: White Blood Cells, Vit D: Vitamin D, PTH: Parathyroid Hormone

Parameter	Patient value	Reference range
WBC	8,420	4,500-11,000/mm^3^
Hemoglobin	13.2 g/dL	13.5-17.5 g/d
Platelets	206,000	150-400x10^9^/L
Creatinine	0.51 mg/dL	<1 mg/dL
Sodium	140 mEq/L	135-145 mEq/L
Potassium	3.7 mEq/L	3.5-5.2 mEq/L
Chloride	113 mEq/L	95-105 mEq/L
Magnesium	1.81 mg/dL	1.4-2.1 mg/dL
Calcium	9.3 mg/dL	8.5-10.2 mg/dL
Vit D	16.6 ng/mL	<30 ng/mL
Phosphorus	1.6 mg/dL	3-4.5 mg/dL
PTH	253.82 pg/mL	10-60 pg/ml

The patient’s symptoms improved following correction of electrolyte abnormalities and optimization of vasodilator therapy. She was discharged on dual calcium channel blockade (verapamil and amlodipine), long-acting nitrates, and appropriate supplementation, with close outpatient follow-up arranged.

## Discussion

Myocardial ischemia does not necessarily result from increased myocardial oxygen demand. In 1959, Dr. Myron Prinzmetal first described “a variant form of angina pectoris” as a distinct entity, differentiating it from the classic angina originally reported by Heberden in 1772, which was typically precipitated by exertion and relieved by rest or nitroglycerin [5]. The incidence of VSA remains uncertain and varies significantly across populations, with a higher prevalence reported in Japanese patients compared with Western populations [6]. CAS may be precipitated by various triggers, including emotional stress, exposure to cold, cocaine use, and heavy smoking. Although its precise pathophysiology remains incompletely understood, mechanisms involving autonomic nervous system imbalance, endothelial dysfunction, chronic vascular inflammation, oxidative stress, and hypercontractility of vascular smooth muscle cells have been implicated. The balance between sympathetic and parasympathetic tone is also an important factor regulating coronary blood flow. Imbalance in this system can predispose to exaggerated vasoconstriction under normal circumstances and in response to agents such as acetylcholine and methacholine [7,8].

In patients with VSA, CAS can be provoked by various stimuli acting through different receptors and intracellular signaling pathways, indicating that the underlying abnormality is more likely situated at a post-receptor, intracellular signaling level. Conventional cardiovascular risk factors show only limited association with CAS, except for cigarette smoking, which is present in approximately 75% of patients with variant angina. Although the precise mechanism by which smoking induces coronary spasm remains unclear, tobacco smoke contains multiple vasoactive and pro-inflammatory components, including nicotine and carbon monoxide, that may trigger spasmogenic alterations in vascular smooth muscle cells in susceptible individuals. Excessive alcohol intake has also been implicated in the development of VSA, although the epidemiological evidence is less robust. In addition, several pharmacological agents and recreational drugs, such as cocaine, amphetamines, marijuana, 5-fluorouracil, capecitabine, and triptans, have been linked to the onset or exacerbation of CAS. Environmental factors, including cold exposure, as well as physiological maneuvers such as Valsalva, hyperventilation, and coronary instrumentation during catheterization, may also precipitate coronary hyperreactivity. Electrolyte disturbances, particularly hypocalcemia and hypomagnesemia, have been reported in association with CAS; in contrast, hypophosphatemia has not been described as a precipitating factor for coronary vasospasm in prior reports. To assess this, a structured literature search was performed using PubMed/MEDLINE and Google Scholar up to October 2025, employing combinations of the terms “hypophosphatemia”, “coronary artery spasm”, “vasospastic angina”, “Prinzmetal angina”, and “MINOCA”. No published cases directly linking hypophosphatemia to angiographically documented CAS were identified [7,8].

CAS most often occurs at rest, particularly during the night and early morning hours. Although Prinzmetal initially emphasized that variant angina was not induced by exercise, it is now recognized that mild exertion in the early morning may provoke coronary spasm, whereas strenuous activity in the afternoon generally does not. This pattern reflects a circadian variation in vascular tone, with epicardial coronary artery constriction peaking in the early morning and relaxing later in the day. Interestingly, this circadian rhythm parallels the timing of ischemic cardiac events, such as acute myocardial infarction and sudden cardiac death, which are also more frequent during early morning hours [9].

Focal CAS, usually due to hyperreactivity of vascular smooth muscle, has been well documented, particularly following the implantation of drug-eluting stents. By contrast, diffuse spasm involving multiple epicardial vessels is less common and is often attributed to widespread endothelial dysfunction. Factors such as cigarette smoking and systemic inflammatory disorders, including rheumatoid arthritis, may contribute to this diffuse endothelial impairment. Importantly, diffuse CAS may mimic fixed obstructive coronary disease and could result in inappropriate revascularization strategies, including PCI or coronary artery bypass grafting (CABG) [10].

To improve diagnostic consistency, the Coronary Vasomotion Disorders International Study Group (COVADIS) has proposed specific diagnostic criteria for VSA. These include relief of spontaneous angina following nitrate administration, transient ischemic electrocardiographic changes during anginal episodes, and angiographic evidence of coronary vasospasm [8]. In the absence of electrocardiographic confirmation, further evaluation is necessary to exclude fixed obstructive CAD. Noninvasive stress testing can be helpful, with most patients showing normal results; however, approximately 10%-30% may develop exercise-induced spasm with ischemic ECG changes that are indistinguishable from fixed obstructive disease. In such cases, coronary angiography is warranted. Similarly, a negative stress test in patients with persistent clinical suspicion should prompt invasive angiographic evaluation [8].

During catheterization, CAS may be observed spontaneously or induced using agents such as ergonovine, acetylcholine, or hyperventilation. While these provocative tests can aid in diagnosis, they are currently less frequently performed. Instead, routine administration of intracoronary nitroglycerin during angiography is recommended to distinguish vasospasm from fixed coronary disease and to prevent unnecessary interventions [8]. Refractory VSA, defined as persistent angina despite the use of maximally tolerated doses of two medications, typically a calcium channel blocker (CCB) and a nitrate, remains a therapeutic challenge. In such cases, and in the absence of standardized guidelines, alternative or unconventional therapies are often employed based on expert opinion to provide symptomatic relief [8,11].

In the emergency setting, the management of VSA generally follows the ACS guidelines, tailored to the presenting clinical scenario. Rapid resolution of chest pain with concurrent normalization of ST-segment elevation after nitroglycerin administration is highly suggestive of coronary spasm. In patients with a known history of VSA, careful review of prior medical records may assist emergency physicians in promptly administering short-acting nitrates and optimizing pharmacological therapy to reduce the risk of recurrence and readmission [12].

First-line therapy for CAS consists of nitrates and CCBs. Isolated β-blocker therapy should be avoided in patients with VSA, as unopposed α-adrenergic activity may exacerbate coronary vasoconstriction in the absence of concomitant CCB use. Long-acting nitrates may be employed as adjunctive prophylactic therapy. Coronary angiography plays a key diagnostic and therapeutic role in the acute setting by enabling direct visualization of coronary spasm and prompt relief with intracoronary vasodilators, such as nitrates or CCBs. This approach is fundamentally distinct from mechanical revascularization. Although most patients achieve adequate symptom control with optimized medical therapy and intracoronary vasodilator administration when required, the role of mechanical revascularization (e.g., PCI) remains controversial. Its use should be restricted to highly selected cases with angiographically documented focal spasm at the site of coexistent fixed coronary stenosis and persistent ischemia despite maximal pharmacological therapy [13].

## Conclusions

This case highlights the importance of broadening the differential diagnosis in refractory vasospastic angina to include electrolyte disturbances, including hypophosphatemia, as potential contributory factors rather than definitive triggers. While established mechanisms such as cigarette smoking and endothelial dysfunction remain central to the pathogenesis of coronary artery spasm, metabolic abnormalities may lower the threshold for vasospastic episodes in susceptible individuals.

Clinically, this supports not only routine electrolyte assessment but also careful evaluation and correction of metabolic derangements as part of a comprehensive management strategy for patients with recurrent or treatment-resistant vasospastic angina. Further observational and mechanistic studies are needed to clarify the role of phosphate metabolism and other electrolyte disturbances in coronary vasomotion and their potential implications for clinical practice.
